# Human Papillomavirus Prophylactic Vaccination improves reproductive outcome in infertile patients with HPV semen infection: a retrospective study

**DOI:** 10.1038/s41598-018-19369-z

**Published:** 2018-01-17

**Authors:** Andrea Garolla, Luca De Toni, Alberto Bottacin, Umberto Valente, Maurizio De Rocco Ponce, Andrea Di Nisio, Carlo Foresta

**Affiliations:** Department of Medicine, Unit of Andrology and Reproductive Medicine, Section of Endocrinology & Centre for Male Gamete Cryopreservation, University of Padova, Padova, Italy

## Abstract

In this study we aimed to evaluate the effect on reproductive outcome of HPV vaccination in male subjects of infertile couples with HPV semen infection. In this single-center study, we retrospectively enrolled 151 infertile couples with detection of HPV in semen, attending our Hospital Unit of Andrology between January 2013 and June 2015, counseled to receive adjuvant HPV vaccination. Seventy-nine accepted vaccination (vaccine group) whilst 72 did not (control group). Our protocol of follow-up, aimed to evaluate HPV viral clearance, consisted in semen analysis, INNO-LiPA and FISH for HPV in semen cells after 6 and 12 months from basal evaluation. Spontaneous pregnancies, miscarriages and live births were recorded. Progressive sperm motility and anti-sperm antibodies were improved in the vaccine group at both time points (p < 0,05 *vs* control arm). Forty-one pregnancies, 11 in the control group and 30 in the vaccine group, were recorded (respectively 15% and 38,9%, p < 0,05) and resulted into 4 deliveries and 7 miscarriages (control group) and 29 deliveries and one miscarriage (vaccine group, p < 0,05 *vs* control group). HPV detection on sperms was predictive of negative pregnancy outcome. Adjuvant vaccination associated with enhanced HPV healing in semen cells and increased rate of natural pregnancies and live births.

## Introduction

Sexually-transmittable diseases are among the primary causes of infertility^1,2^. In this context, despite genital human papillomavirus (HPV) is acknowledged as the most common sexually-transmitted viral infection worldwide, very few studies have investigated the effect of HPV on human reproduction. Data on the actual rate of spontaneous abortions, major birth defects and pregnancy complications during natural conceptions in couples exposed to HPV appear scarce and controversial^3,4^. Dealing with assisted reproduction, only a clinical study performed on women undergoing *in-vitro* fertilization (IVF) reported a significant reduction of pregnancies in the presence of cervical HPV detection^5^.

New recent insights on the role of HPV in human reproduction derived from studies on infertile couples with viral infection detected in semen. Indeed, the prevalence of HPV-DNA detection in semen from infertile males is nearly 3 to 4 folds higher than fertile controls^6,7^. Even in male patients with accessory gland infection the prevalence of HPV detection in semen is 2 to 3 to folds higher than healthy subjects, regardless of the mere inflammatory or the microbial form of the disease^8^. Furthermore, prevalent sperm motility impairment and detection of anti-sperm antibodies (ASA) have been described in male subjects with detection of HPV-DNA in semen^9–11^.

From a mechanistic point of view, the effect exerted by HPV infection in semen during the fertilization process is currently under investigation. To this regard, data from our group showed that sperm cells, either transfected with HPV E6/E7 genes or exposed to HPV L1 capsid protein, were able to penetrate the oocyte and to transfer the virus DNA into oocytes where viral genes were then activated and transcribed^12^. As a consequence, a possible role of sperm cells as vectors for HPV transfer into the oocytes could be suggested^12^. To this regard, the possible consequences of embryo exposure to HPV are not well defined. However *in vitro* experiments have shown that trophoblast cells transfected with HPV-DNA have an increased rate of stage-specific maturation arrest, apoptosis and a reduced placental invasion into the uterine wall, compared with controls^13^. These data are largely in agreement with the few available clinical evidence showing an association between the detection of HPV-DNA in spermatozoa and reduced pregnancy rate or recurrent miscarriage^14–18^.

The prophylactic vaccination anti-HPV is demonstrated to be highly effective in preventing HPV-related pathologies in both sexes, such as cervical lesions, vulvo-vaginal lesions, condylomas in females^19–26^ together with anal pre-cancerous lesions, external genitalia lesions and pharyngeal pathologies in males^27–29^.

However, despite the overall genital HPV infection is highly prevalent among all age groups of men, the HPV vaccination coverage in eligible males is less than 11%^30^.

We recently described that HPV vaccination in males with HPV detection in semen, led to a significant reduction of the prevalence of HPV semen infection together with amelioration of sperm parameters, such as motility and anti-sperm antibodies (ASA), through the likely stimulation of humoral immunity^31^. This evidence arises questions about the possible application of prophylactic vaccination in male patients whose couple infertility is likely related to the tresence of HPV in semen^32,33^. On these bases, here we aimed to evaluate the effect of HPV vaccination on reproductive outcome, in terms of natural pregnancy, delivery and abortion rate. To this aim, we retrospectively evaluated the pregnancy outcome in the 12 months-follow up of infertile couples where the male partner showed HPV semen infection and received the HPV prophylactic vaccination. A group of infertile couples with equal HPV-detection in semen that did not receive vaccination served as control group. We also evaluated the effect of HPV vaccination on viral persistence and semen parameters, particularly in terms of motility and presence of ASA in both groups.

## Results

**Figure 1:**
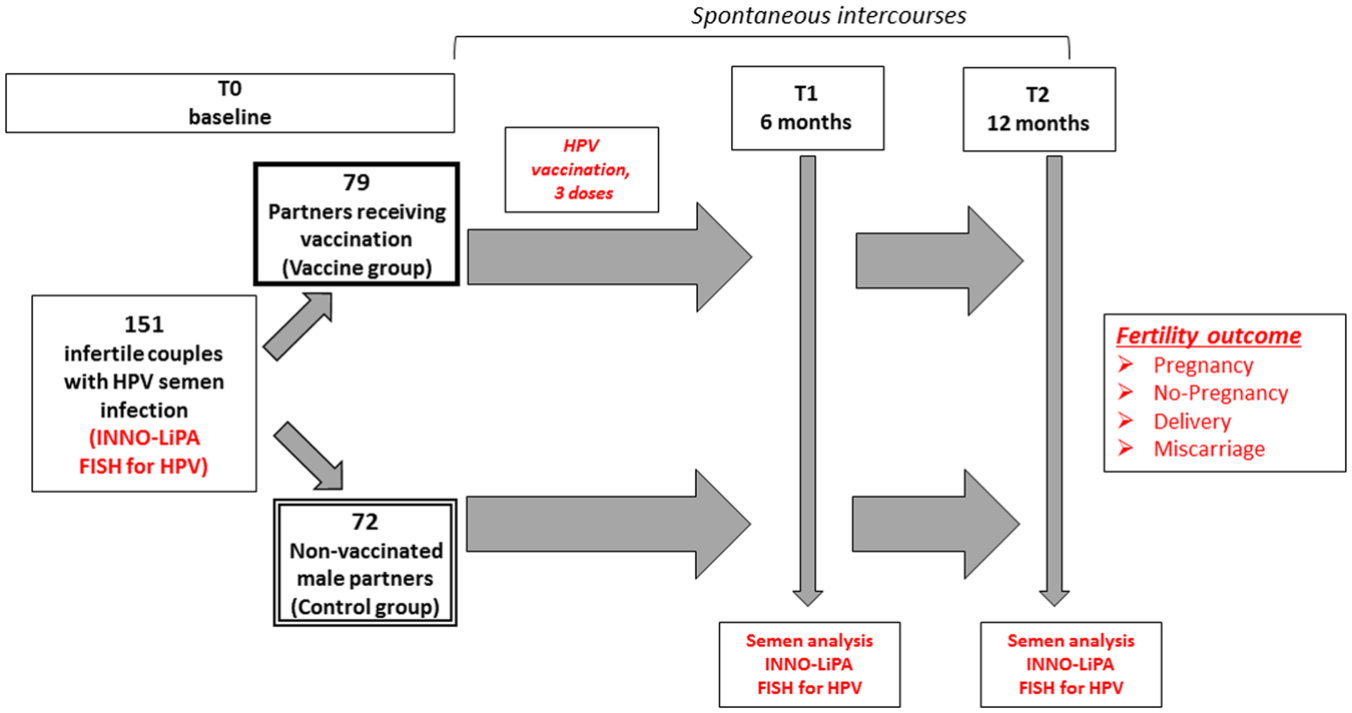
Flow diagram of the study showing the sample size of the study cohort, the proportion of patients accepting vaccination and the time-points for the clinical evaluation of viral healing.

As summarized in Fig. 1, a total of 151 couples, respectively 79 in the vaccine group and 72 in the control group, were considered for the analysis. Table 1 shows the clinical characteristics of male partners as a whole and divided according to the intervention received. The pattern of semen parameters substantially overlapped with that of previous studies on infertile patients with detection of HPV-DNA in semen^12^. No significant difference was observed between control and vaccine groups in terms of clinical characteristics. The cumulative frequency of each HPV genotype, detected by INNO-LiPA, in the whole cohort of patients at baseline is reported in supplementary Figure S1. No difference in the distribution of HPV genotypes was observed comparing the two groups (data not shown).

**Table 1:** Clinical characteristics of infertile patients with HPV semen infection as a whole group and divided by vaccine treatment.

	All patients(N = 151)	Control group (N = 72)	Vaccine group (N = 79)
	Mean ± SD	Mean ± SD	Mean ± SD
Age (years)	32.6 ± 3.0	32.8 ± 2.8	32.4 ± 3.1
Total sperm (cells x 10^6^)	173.9 ± 149.2	182.6 ± 128.1	165.1 ± 163.9
Progressive motility (%)	31.9 ± 15.2	32.3 ± 15.1	31.6 ± 15.5
Normal morphology (%)	11.3 ± 6.7	11.7 ± 5.8	10.2 ± 6.9
Sperm antibodies (%)	11.0 ± 21.5	12.4 ± 23.4	9.8 ± 20.3

### Semen parameters

**Figure 2:**
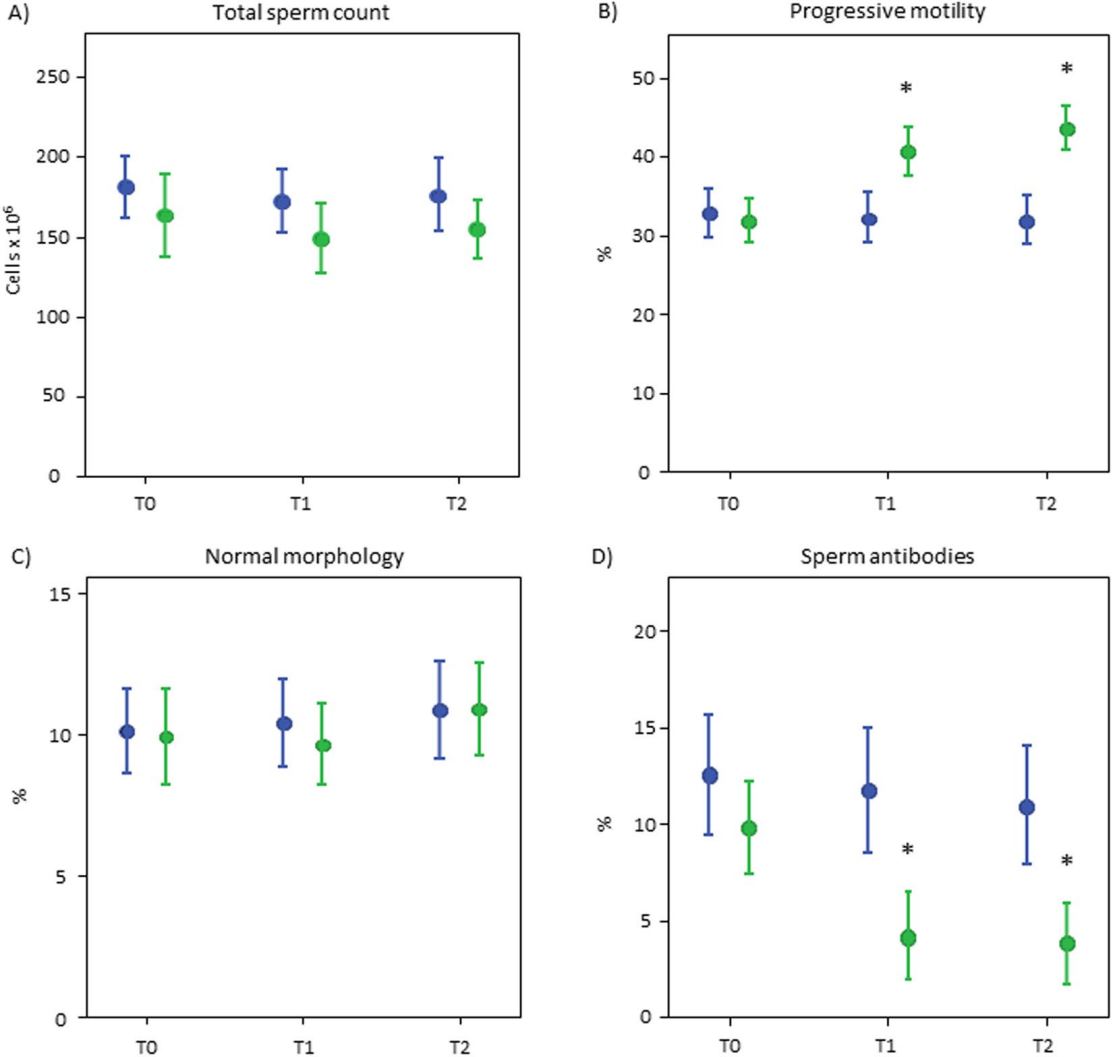
Means (±standard errors) of total sperm count (**A**), progressive motility (**B**), normal morpholoy (**C**) and anti-sperm antibodies (**D**) observed in the control arm (blue bars) and in the vaccine arm (green bars) at the different time-points. T0: Baseline; T1: 6 months (at the end of HPV vaccination); T2: 12 months (after further 6 months from the end of HPV vaccination). *p < 0.05 vs control arm.

Figure 2 shows the mean values with standard errors of semen parameters observed at different time-points of the follow-up in the control and in the vaccine groups. Compared to controls, a significant improvement of progressive sperm motility and anti-sperm antibodies was observed in the vaccine group (all p values < 0.05 *vs* corresponding time point of the control arm). This change was present at T1 (6 months, corresponding to the end of the treatment period) and was still present at T2 (12 months, corresponding to 6 months after the end of HPV vaccination). No significant modification was observed in total sperm count and normal morphology.

Table 2 shows the prevalence of HPV-DNA detection, by either INNO-LiPA or FISH analysis, and anti-sperm antibodies in the two groups at the different time-points of the follow-up. Results of FISH analysis are reported as raw numbers and percentage of patients with any positive staining for HPV-DNA either sperm cells, exfoliated cells (E.c.) or both. From T0 to T2 there was a progressive and significant reduction of patients with HPV-DNA infection in both groups. In particular, T1 and T2 respectively, the percentage of infected subjects in the control group was 86.1% and 70.8%, whereas in the vaccine group was 30.4% and 10.1% at (p < 0.05 vs control arm at both tome-points). Considering the FISH analysis, a significant reduction of the percentage of patients showing a positive staining for HPV-DNA in sperm cells was observed in both control and vaccine groups (p < 0.05 for both groups vs T0), although the decrement was stronger in the latter group of patients (p < 0.05 vs control group at both time-points). A reduction of the percentage of patients with positive HPV staining on E.c. or on both sperm and E.c. was observed at T1 and T2 only in the vaccine arm (all p < 0.05 vs T0 and vs control group). In parallel, a significant reduction of the percentage of patients with anti-sperm antibodies was observed only in the vaccine group (Table 2).

**Table 2:** Prevalence of HPV infection, detected by INNO-LiPA and FISH, and anti-sperm antibodies in the control arm and vaccine arm at different time-points.

		Patients with + HPV-DNA N (%)	Patients with + FISH on Sperm N (%)	Patients with + FISH on E.c. N (%)	Patients with + FISH on Sperm and E.c. N (%)	Patients with + Anti-sperm antibodies N (%)
Control Arm	T0	72 (100)	64 (88.9)	43 (59.7)	36 (50)	28 (38.9)
	T1	62 (86.1)^b^	51 (70.8)^b^	42 (58.3)	36 (50)	28 (38.9)
	T2	51 (70.8)^bc^	47 (59.5)^b^	39 (54.1)	32 (44.4)	22 (30.6)
Vaccine Arm	T0	79 (100)	68 (86.1)	51 (64.5)	41 (51.2)	35 (44.3)
	T1	24 (30.4)^ab^	24 (30.4)^ab^	19 (24.1)^ab^	15 (19)^ab^	13 (16.4)^ab^
	T2	8 (10.1)^abc^	7 (8.9)^abc^	8 (10.1)^abc^	6 (7.6)^abc^	25 (31.6)^abc^

Data for FISH analysis are presented as positivity of sperm cells, exfoliated cells (E.c.) or both. T0: baseline; T1: 6 months (at the end of HPV vaccination); T2: 12 months (after further 6 months from the end of HPV vaccination).

^a^p < 0.05 *vs* respective time-point in the control arm

^b^p < 0.05 *vs* T0

^c^p < 0.05 *vs* T1.

**Figure 3:**
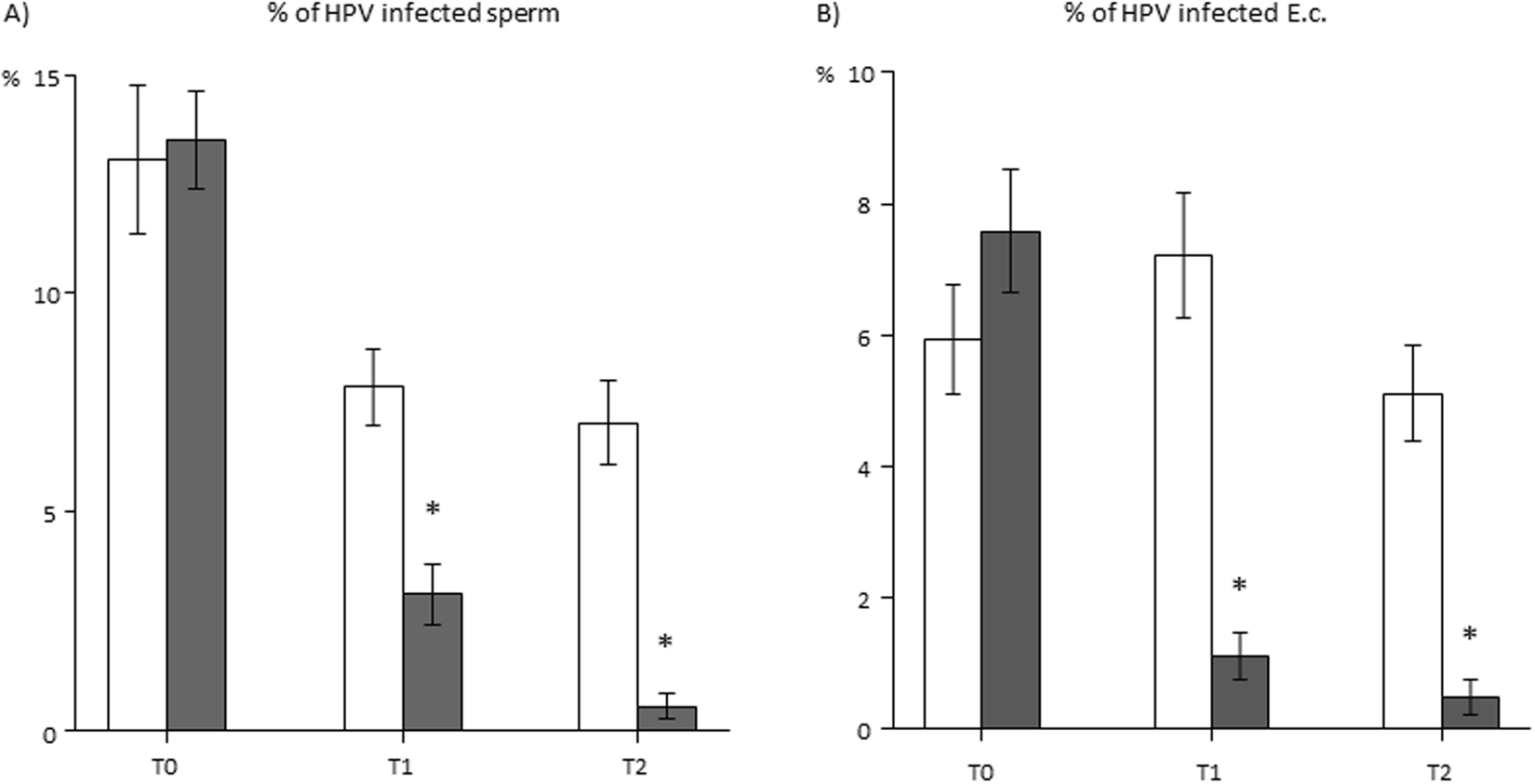
Mean percentage (±standard errors) of (**A**) HPV-infected sperm and (**B**) exfoliated cells (E.c.), observed by FISH analysis, in the control arm (empty bars) and in the vaccine arm (grey bars) at different time points. T0: Baseline; T1: 6 months (at the end of HPV vaccination); T2: 12 months (after further 6 months from the end of HPV vaccination). *p < 0.05 vs control arm.

In Fig. 3, panels A and B show respectively the mean percentage of sperm and E.c with positive staining for HPV-DNA, observed by FISH analysis in the control and in the vaccine groups at different time points. In the vaccine group a significant reduction of both infected sperm and E.c at T1 and T2 was observed. This evidence was not present in the control group (both p < 0.05 vs control group). Supplementary Table 1 shows the distribution of semen parameters on the basis of negative or positive results, at FISH analysis in sperm cells, E.c. or both, in patients from control and vaccine groups at the end of the study. In both control and vaccine groups, progressive motility resulted significantly lower when HPV infection was detected in sperm cells (p < 0.05 vs negative subjects). Independently of the cellular localization of HPV, progressive motility was significantly higher in FISH-negative patients from the vaccine arm (p < 0.05 vs. control group). As for progressive motility, the presence of ASA was significantly associated to HPV infection at sperm level in both control and vaccine arms (p < 0.05 vs negative subjects). Again, the presence of ASA was significantly lower in vaccine arm, independently of the localization of HPV at sperm or E.c. level only in FISH-negative patients (p < 0.05 vs. control group).

### Pregnancy outcome

**Figure 4:**
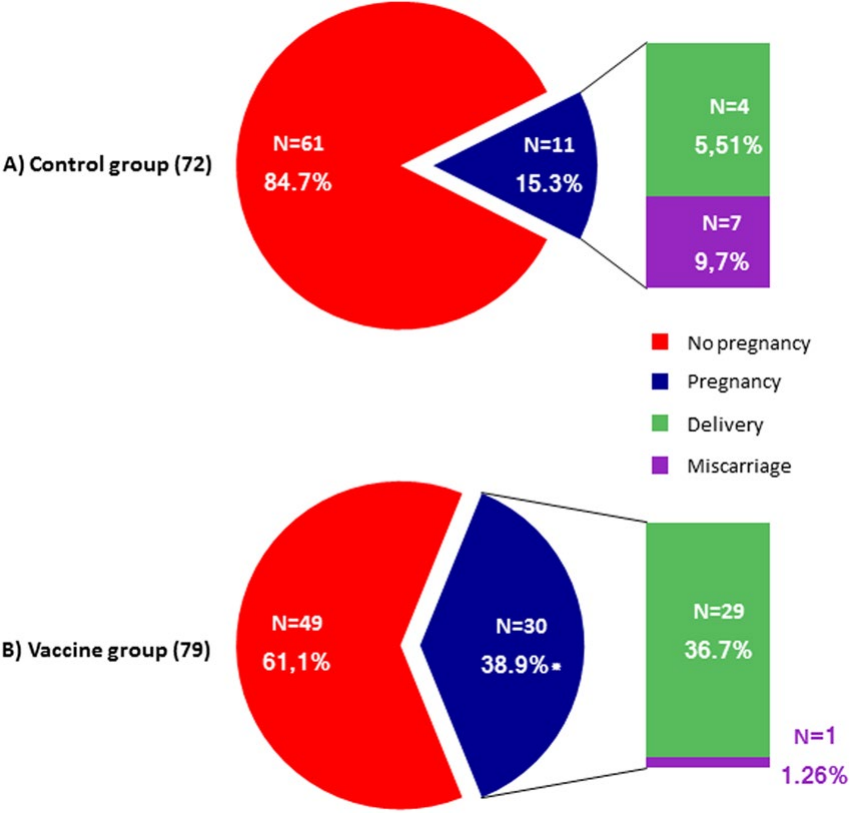
Proportion of natural pregnancies (blue), deliveries (green) and abortions (purple) at T2 from couples of the control arm (**A**) and vaccine arm (**B**). *p < 0.05 vs control arm.

The pregnancy rate and the pregnancy outcome observed in couples from control and vaccine groups are reported in Fig. 4. At T2, overall 41 couples achieved a pregnancy, respectively 11 in the control group (15.3%) and 30 in the vaccine group (38.9%, p < 0.01 *vs* control group). Among couples of the former group, 4 deliveries and 7 miscarriages were recorded whereas, among couples of the vaccine group, we recorded 29 deliveries and one miscarriage. The control group also showed a higher miscarriage rate than the vaccine group (p < 0.05, Fig. 4). In particular, all pregnancy losses in the control group occurred very early during the follow-up period (respectively, one at 5th and two at 7th gestational week). Furthermore, we observed a different localization of HPV semen infection was different in those cases that recorded deliveries or miscarriages. In particular, the 4 cases with deliveries showed no semen infection or infection confined to E.c., whilst the 7 cases with miscarriage always showed the presence of infected sperm (two cases in both sperm and exfoliated cells and one case confined to sperm). The only miscarriage observed in the control arm took place at the 6th gestational week, and the male partner had HPV semen infection confined to E.c.

Table 3 reports sperm parameters and results of HPV-DNA detection in sperm or E.c by FISH analysis, observed at T2 in patients who achieved natural pregnancy or not in the two groups. Subjects of the control group who achieved pregnancy during the study period had higher sperm count, progressive motility and lower levels of HPV-DNA detection in sperm cells (all p < 0.05 vs no pregnancy). All subjects of the vaccine group (pregnancy and no pregnancy) had higher progressive motility and reduced levels of both anti-sperm antibodies and sperm/E.c. with positive staining for HPV-DNA (all p < 0.05 vs control arm). Moreover, among subjects of the vaccine group, those who achieved pregnancy had significantly lower levels of positive staining for HPV-DNA on sperm cells compared to those that achieved no pregnancy (Table 3).

**Table 3:** Sperm parameters and FISH analysis for HPV, observed at the end of the study in patients who achieved natural pregnancy, or not, from the control arm and vaccine arm.

		Sperm count (cells x 10^6^)	Progressive Motility (%)	Normal morphology (%)	Anti-sperm antibodies (%)	HPV sperm infection (%)	HPV E.c. infection (%)
Control arm (N = 72)	No pregnancy (N = 61)	106.3 ± 52.5	29.9 ± 12.4	11.0 ± 6.1	11.2 ± 19.9	7.8 ± 5.6	5.6 ± 5.1
	Pregnancy (N = 11)	188.1 ± 99.9^b^	35.2 ± 10.8^b^	9.6 ± 6.2	7.6 ± 18.8	1.6 ± 4.0^b^	2.8 ± 7.2
Vaccine arm (N = 79)	No pregnancy (N = 49)	143.6 ± 124.2	42.3 ± 9.1^a^	11.0 ± 6.1	5.4 ± 9.1^a^	1.2 ± 2.3^a^	0.6 ± 2.2^a^
	Pregnancy (N = 30)	168.7 ± 140.2	46.7 ± 13.7^a^	10.6 ± 6.4	0.9 ± 3.2^a^	0^ab^	0.3 ± 1.1^a^

Data for FISH analysis are presented as positivity of sperm cells or exfoliated cells (E.c.).

^a^p < 0.05 vs control arm

^b^p < 0.05 vs. no pregnancy.

## Discussion

In this retrospective study we compared natural fertility in idiopathic infertile couples with naive and vaccinated male partners with detection of HPV-DNA in semen. Our findings, besides confirming the negative influence of semen HPV infection on sperm motility and ASA, showed for the first time that HPV vaccination in infected males associates with increased pregnancy rate and delivery rate of healthy born babies together with a lower rate of miscarriages. In particular, vaccinated patients had improved sperm motility, reduced levels of ASA and a lower detection of HPV on both sperm and E.c. Moreover, the most predictive parameter of pregnancy outcome and delivery rate was represented by the absence of HPV on sperm, since none of the male patient from couples that achieved live birth showed a positive staining for HPV-DNA on sperm. On the other hand, we observed that all miscarriages reported in the control arm were associated with a positive staining of HPV-DNA on sperm cells. On these bases it might be speculated that, contrarily to E.c., detection of HPV-DNA on sperm is poorly relevant for the viral life cycle, but valued for the role of sperm as passive carriers of virus attached to the cell surface as postulated for other viral infections^34^.

The state of the art concerning HPV sperm infection and infertility, indicates that: i) there is a higher prevalence of HPV semen infection in men affected by idiopathic infertility compared with the general population, independently from the genotype of HPV detected; ii) in infected infertile patients, HPV is mainly localized on the sperm surface, in particular along the equatorial region; iii) there is a significant relationship between asthenozoospermia and HPV sperm infection; iv) the detection of HPV-DNA on sperm surface is more frequently associated with the detection of ASA and this condition is characterized by a further reduction of sperm motility; v) *in vitro* evidence shows that, when HPV is bound to spermatozoa, it is likely transferred to fertilized oocytes leading to an impairment of embryo development into blastocysts, and trophoblast cells; vi) recent evidence reported a possible role of HPV sperm detection in assisted reproduction failure and adverse pregnancy outcome (reviewed in^14,15^ and^33,35^). However, the actual role of HPV semen infection in couples seeking natural fertility receive less interest.

The available HPV prophylactic vaccine demonstrated high efficiency in preventing viral infection and HPV-related diseases^19–29^. However, healing effects of neutralizing antibodies against recombinant self-assembled virus-like particles were early suggested in animal models^36–38^. In humans, a recent systematic review showed that when HPV vaccination is used as an adjuvant treatment for active clinical disease, a decreased disease burden is observed^39^. Accordingly, we recently reported that HPV prophylactic vaccination improves viral healing and sperm parameters by involving humoral immunity^31^. To this regard, a recent study from our group on infertile couples eligible for assisted reproduction showed a reduced pregnancy rate and a significantly higher abortion rate when the male partner had positive FISH analysis on sperm^16^. Based on these evidence, we suggested that HPV vaccination may represent a valuable tool to improve viral healing in infertile couples, aimed to improve reproductive outcome. It should also be noted that HPV vaccination itself does not increase the risk of miscarriage in pregnancy, as a long-term observational study has recently confirmed^40^. As in previous studies, we observed that patients with HPV-DNA on sperm at FISH analysis showed a positive staining in a relatively small percentage of cells. Major concerns may then derive from the fact that patients, despite this pattern of infection, have worse pregnancy rates and fail to achieve live births. To this regard, we hypothesize that the lack of HPV detection in the remaining sperm population is likely related to the small viral DNA quantity, lower than method’s sensitivity^41^.

The main limitation of this study is represented by the relatively small sample size in relation to the wide range of HPV genotypes detected and the use of a vaccine whose specificity is restricted to only four genotypes. Indeed, the effect of HPV in male infertility was shown to be largely independent from viral genotype^9,11^ and, accordingly, our patients showed a wide variety of types from low to intermediate and high risk. However, it is recognized a degree of cross-protection provided by quadrivalent vaccine^21,41^. In addition, as reported for general population, in infertile patients, and in those here described, HPV infection is mainly ascribed to both the types covered by the vaccine and those defined by cross-protection^31^.

Considering the wide prevalence of HPV infection in infertile couples, these results may directly impact on their diagnostic work-up, being supportive of a screening for HVP even at the very beginning of this process. In case of viral DNA detection in semen, FISH analysis for HPV would address the diagnostic accuracy of infection and possibly the convenience of a therapeutic approach with vaccination^33^.

In conclusion, HPV screening in infertile male patients is strongly recommended and we suggest to perform FISH analysis in infected subjects, in order to ascertain the pattern of the viral infection. Since prophylactic vaccination enhances healing from HPV infection in semen cells, we propose HPV vaccination as a suitable strategy to improve assisted and even natural fertility.

## Materials and Methods

We conducted a retrospective study on infertile couples who attended the Unit of Andrology and Reproductive Medicine between January 2013 and September 2016. At recruitment, all couples reported the seek for fertility since at least one year and gave written informed consent for the access to their clinical information and the use of their data according to the Italian Privacy Law. The study was approved by the Ethical Committee for Clinical Experimentation of the Padova University Hospital, Protocol number 2336 P and subsequent amendments.

Among infertile couples, those eligible for recruitment had idiopathic infertility and detection of HPV-DNA in semen of the male partner. At the time of diagnosis, infected patients received medical counseling, including HPV vaccination aimed to accelerate viral clearance, as previously described^33,42^. Patients who agreed HPV vaccination, received off-label prescription of the quadrivalent vaccine Gardasil (Merck Serono S.p.A., Milan, Italy). Vaccine was administered as 3 injections over 6 months, the second dose received 2 months after the first dose, and the third dose received 6 months after the first dose respectively. The procedure was performed at the Unit of Hygiene and Public Health of Padova Hospital after acceptance of written informed consent.

Semen analysis, ASA detection, INNO-LiPA for HPV detection/genotyping and FISH for HPV-DNA on sperm and exfoliated cells (see below) were evaluated in all male patients throughout an overall follow-up of 1 year. Analyses were performed at baseline (T0), at the end of HPV vaccination (6 months from baseline, T1) and after further 6 months from the end of HPV vaccination (12 months from baseline, T2). During the study period, clinical pregnancy rates (by positive serum β-hCG test), ongoing pregnancy rates (uncomplicated pregnancy over 12 gestational weeks), miscarriages and healthy born babies were also recorded. The prevalence of HPV infection in semen and pregnancy outcomes during the whole study period, were retrospectively evaluated in patients that received vaccination (the “vaccine group”) and in non-vaccinated patients (the “control group”).

### Partecipants

As male partners eligible for the study were considered subjects aged 25 to 40 years with HPV semen infection and total sperm count ≥40 × 10^6^ cells per ejaculate, independently from the other sperm parameters evaluated according to World Health Organization guidelines 2010^43^. Exclusion criteria were: concomitant infection of *Chlamydia trachomatis*, *Ureaplasma urealyticum*, *Neisseria gonorrhoae* or other sperm infections, together with seropositivity toward human immunodeficiency virus type 1 or 2, human T-cell lymphotropic virus type 1 or 2, hepatitis B or C virus and *Treponema pallidum*. Patients with karyotype abnormalities or CFTR mutations were also excluded. Screening and genotyping for HPV-DNA together with analysis of localization of HPV on spermatozoa and/or exfoliated cells were performed on whole semen respectively by INNO-LiPA Genotyping and Fluorescent *in situ* hybridization (FISH) as previously described^6,31^. Multiple HPV infections at recruitment were defined as the detection of ≥2 different HPV types in whole semen by the genotyping assay.

Dealing with the female partner, were considered as eligible normo-ovulatory women with the following characteristics: normo-responder according to Bologna Criteria^44^ idiopathic/unexplained infertility, age between 25 and 35 years, BMI between 18 and 30, negative pap smear and genital swab for the presence of *Chlamydia trachomatis*, *Neisseria gonorrhoae*, *Trichomonas vaginalis*, bacterial vaginosis. Patients with previous ovarian/tubal surgery, patients with a previous history of cervical dysplasia, positive history for endometriosis, pelvic inflammatory disease, tubal occlusion and polycystic-ovarian-syndrome were exluded. In addition, patients treated for benign endouterine disease (such as endometrial polyps, sub-mucous myomas, intrauterine synechiae and/or uterine septus) in the previous 6 months were excluded. Patients with history of smoking, karyotype abnormalities, mutations of the cystic fibrosis gene, major systemic disease (such as diabetes, multiple sclerosis, adrenal diseases, thyroid dysfunction, alteration in basal serum prolactin value, hypogonadotropic or hypergonadotropic hypogonadism, acquired or inherited thrombophilia and immunological disorders) previous neoplasia, previous chemo and/or radio therapy, untreated uterine diseases (polyps, myomas, synechiae, septus) were also excluded.

### Semen Processing and Anti-sperm antibody detection

Semen samples were collected from all the study partecipants by masturbation. Patients were required to attend the visit with 3 days of sexual abstinence. Semen samples were let liquefy at room temperature and evaluated according to the World Health Organization guidelines for semen analysis^43^. Samples were assessed for semen volume, pH, sperm concentration, viability, motility, and normal morphology. SpermMar Test kit was also applied to detect the presence of anti-sperm antibodies of the IgG and IgA class according to the manufacturer’s protocol (FertiPro N. V., Sint-Martens-Latem, Belgium)^45,46^. Complete coverage of sperm cells by latex particles was considered as positive result for the test whilst the presence of uncovered and moving sperms was considered as negative.

### HPV-DNA screening-genotyping and fluorescence *in-situ* hybridization (FISH)

HPV-DNA screening-genotyping was performed with INNO-LiPA HPV Genotyping Extra assay (Innogenetics, Fujirebio Italia S.r.l., Pomezia, Italy) according to the manufacturers’ protocols as previously reported by Barzon and collegues^47^. Briefly, total DNA isolated form semen underwent PCR amplification using the INNO-LiPA HPV Genotyping Extra assay reagents. Biotinylated PCR products were genotyped by hybridization to HPV type-specific oligonucleotide probes, bound to nitrocellulose membrane, and detected by colorimetric reaction using an Auto-LiPA 48 instrument in accordance with the manufacturer’s recommendations. All results were confirmed by visual inspection by trained personnel.

Glass slides containing at least 2 × 10^6^ smeared sperm, fixed with a methanol-acetic acid solution, were used for FISH analysis for HPV as previously described^48^. Briefly, permeabilized samples were pre-treated with pepsin and incubated with hybridization solution (Pan Path, B.V., Budel, The Netherlands) containing biotin-labeled HPV DNA probe. After denaturation of target DNA, hybridization with HPV-DNA probe was performed by incubating the samples at 37 °C overnight in a humidified chamber. The biotin-labeled HPV probe was detected by incubation with streptavidin Texas Red (Vector Laboratories) at the proper dilution. Slides were mounted with an anti-fade buffer solution containing DAPI as counterstainer (BioBlue; BioView). Samples were analyzed using a fluorescence microscope (Nikon ViCo video confocal microscope, Firenza, Italy) equipped with a triple band-pass filter set (FITC, TRITC, DAPI). For each slide, at least 200 spermatozoa and 200 exfoliated cells (E.c.) were analyzed by three independent investigators and results were reported as the percentage of sperm or E.c. showing a positive staining as described. Supplementary Figure S2 shows examples of FISH analysis for HPV on semen samples of infertile patients.

### Patient involvement

No patients were involved in setting the research question or the outcome measures, nor were they involved in developing plans for design or implementation of the study. No patients were asked to advise on interpretation or writing up of results.

### Statistical Analysis

Statistical analysis of the data were conducted with SPSS 21.0 for Windows (SPSS, Chicago, IL). The results are expressed as means ± standard deviation (SD) and categorical variables are expressed as a percentage. The Kolmogorov–Smirnov test was used to check for normality of distribution. Variables not showing normal distribution were log-transformed. Characteristics of subjects between the control and vaccine arm at each time point were compared by using unpaired Student’s t test. Repeated-measures ANOVA was performed to test for differences in continuous variables during the study at three time points (baseline, 6 months, 12 months). Age was included as a covariate and post-hoc analysis with Bonferroni-Holm correction for multiple comparisons was performed to test differences between time-points. The Levene’s test was used to test the homogeneity of variance among groups. If homogeneity of variance assumption was violated, Welch test was performed and the respective *p* value was reported. The proportion of HPV-positive patients during the study was compared with non-parametric Cochran’s test. Post-hoc pairwise comparisons were performed with McNemar’s test. Differences between groups in categorical variables at each time point were compare with Pearson’s Chi-square test, or Fisher’s exact test when expected frequency was five or less. p values < 0.05 were considered as statistically significant.

## Electronic supplementary material

Supplemental Information
