# Supplementary material for: Human Papillomavirus Prophylactic Vaccination improves reproductive outcome in infertile patients with HPV semen infection: a retrospective study
Source: Sci Rep. 2018 Jan 17;8:912. doi: 10.1038/s41598-018-19369-z (PMC5772512; doi:10.1038/s41598-018-19369-z)
Supplement: Supplementary file 1 — Supplemental Information [file 41598_2018_19369_MOESM1_ESM.pdf]

Human Papillomavirus Prophylactic Vaccination improves reproductive outcome in infertile patients with HPV semen infection: a retrospective study

Authors
Andrea Garolla[1#], Luca De Toni[1#], Alberto Bottacin[1], Umberto Valente[1], Maurizio De Rocco Ponce[1], Andrea Di Nisio[1] & Carlo Foresta[1]*.

[1] Department of Medicine, Unit of Andrology and Reproductive Medicine, Section of Endocrinology & Centre for Male Gamete Cryopreservation, University of Padova, Italy

[#]: The two authors equally contributed to the study

Running title: HPV male vaccination and natural fertility

*Corresponding author:
Prof. Carlo Foresta
Department of Medicine
Unit of Andrology and Reproductive Medicine
University of Padova, Via Giustiniani, 2, 35128 Padova, Italy
Tel +39-49-8218517
Fax +39-49-8215820
e-mail: carlo.foresta@unipd.it

The corresponding author affirms that the manuscript is an honest, accurate, and transparent account of the study being reported. No important aspects of the study have been omitted.

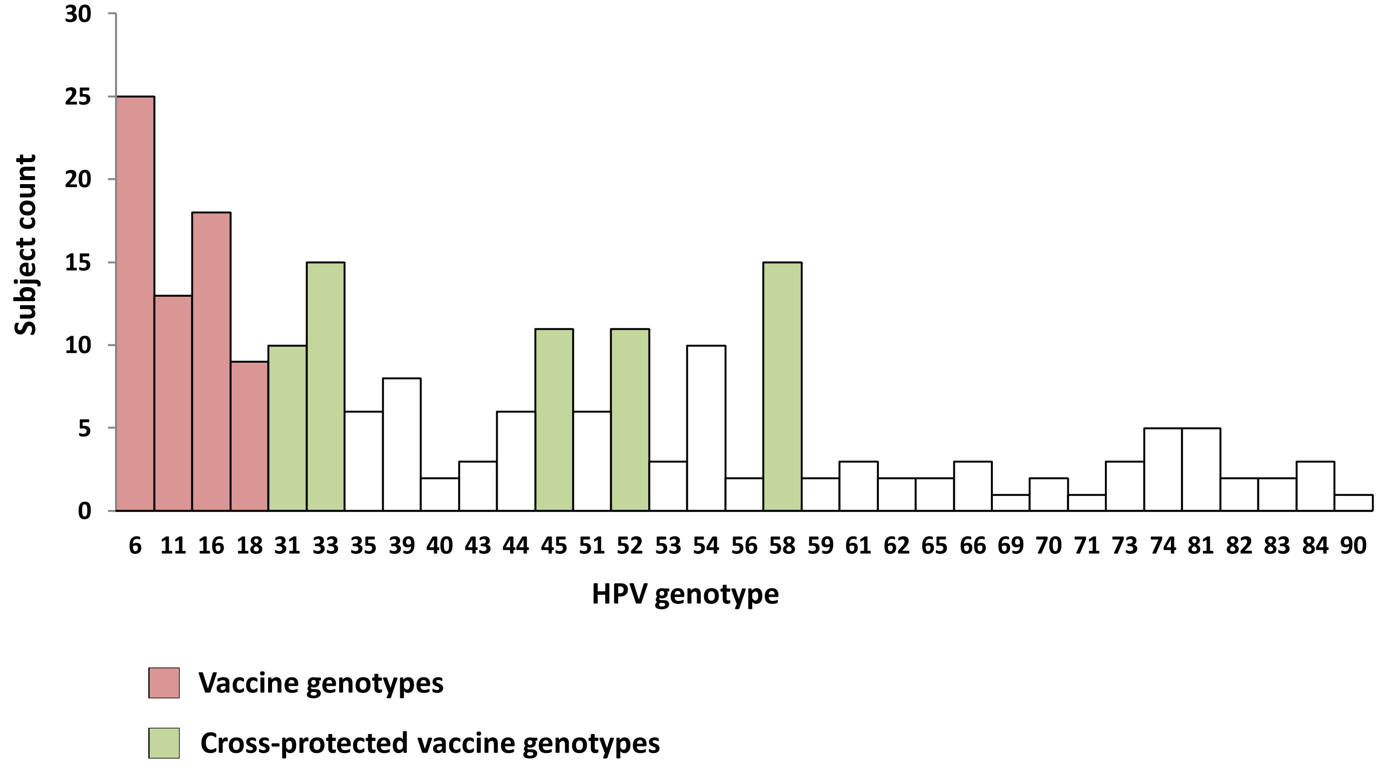

Supplementary Figure S1

Supplementary Table 1. Semen parameters in control and vaccine arm patients. at the end of the study. based on FISH HPV detection in sperm cells. exfoliated cells (EC) and both.

		Sperm HPV (N=151)		EC HPV (N=151)		Sperm + EC HPV (N=151)	
Seminal parameter	Vaccine	Negative (N=97)	Positive (N=54)	Negative (N=104)	Positive (N=47)	Negative (N=113)	Positive (N=38)
Total sperm cells x 10^6	No	146.6 ± 100.4	196.9 ± 105.2	165.5 ± 118.2	191.5 ± 87.5	155.9 ± 111.9	168.4 ± 83.1
	Yes	153.4 ± 129.4	148.1 ± 92.0	159.8 ± 130.2	118.7 ± 77.6	156.1 ± 130.4	122.8 ± 62.1
Motility (%)	No	36.7 ± 12.2	25.5 ± 10.4[a]	34.6 ± 12.1	32.3 ± 10.1	34.7 ± 14.1	27.3 ± 12.2[a]
	Yes	45.9 ± 10.5[b]	30.7 ± 9.3[a]	42.4 ± 10.8[b]	34.8 ± 9.9	44.5 ± 11.5[b]	30.2 ± 11.0[a]
Normal morphology (%)	No	10.9 ± 5.5	10.6 ± 6.0	10.4 ± 6.4	11.2 ± 6.2	9.6 ± 6.2	12.8 ± 6.1
	Yes	10.8 ± 6.1	12.0 ± 7.1	11.1 ± 6.6	8.6 ± 2.7	11.0 ± 6.5	9.0 ± 2.9
Semen antibodies (%)	No	5.6 ± 10.8	18.8 ± 26.4[a]	11.6 ± 25.2	10.2 ± 21.7	5.5 ± 11.3	14.3 ± 28.0[a]
	Yes	3.7 ± 16.1[b]	5.0 ± 5.8	3.6 ± 16.1[b]	6.0 ± 8.2	3.8 ± 15.4	3.5 ± 7.0[b]

Data are reported as means ± standard deviations

[a]= p<0.05 vs negative subjects

[b]= p<0.05 vs. no vaccine

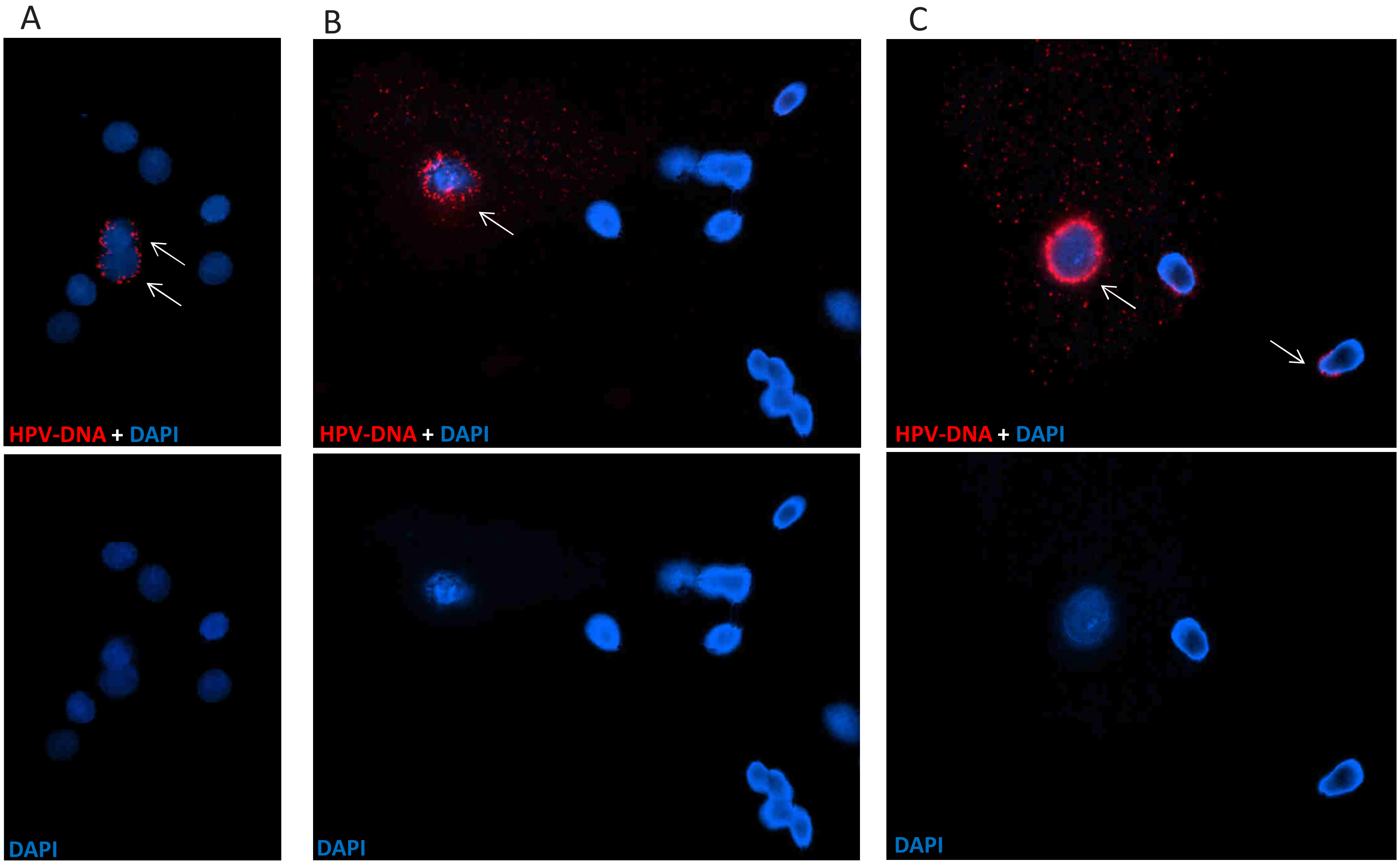

Supplementary Figure S2
